# Long-Term Health Improvements and Economic Performance Among Individuals With Diabetes

**DOI:** 10.1001/jamahealthforum.2025.0756

**Published:** 2025-05-16

**Authors:** Jack M. Chapel, Dana P. Goldman, Matthew E. Kahn, Bryan Tysinger

**Affiliations:** 1 Leonard D. Schaeffer Center for Health Policy and Economics, Los Angeles, California; 2 Sol Price School of Public Policy, University of Southern California, Los Angeles; 3 Department of Economics, Dana and David Dornsife College of Letters, Arts, and Sciences, University of Southern California, Los Angeles

## Abstract

**Importance:**

Advances in diabetes detection and treatment have mitigated the risks of serious complications and death, but little is known about whether economic outcomes for people with diabetes have similarly improved.

**Objective:**

To assess whether associations between diagnosed diabetes and labor market outcomes have changed over time.

**Design, Setting, and Participants:**

This cross-sectional study analyzed data from the National Health Interview Survey from 1998 to 2018. The sample was nationally representative of the US population aged 40 to 64 years. Average marginal effects, the regression-adjusted difference in probability of outcomes between people with and without diabetes, pooled by 3-year periods (1998-2000 to 2016-2018), were estimated with controls for demographics, education, and comorbid health risks. Behavioral Risk Factor Surveillance (BRFSS) data from 1993 to 2023 were included in robustness analyses. Data were analyzed from September 2023 to November 2024.

**Exposure:**

Diagnosed diabetes, defined based on respondents’ self-report that they have ever been diagnosed by a medical professional.

**Main Outcomes and Measures:**

The main outcomes were labor force participation and any Supplemental Security Income or Social Security Disability Insurance income receipt. Secondary outcomes included reporting health limitations for any activities, health limitations for work, any nights in hospital, and receiving health care 10 or more times in the past year.

**Results:**

The study included 249 712 individuals, 25 177 with diabetes. The weighted population was 50% female, 12% Hispanic, 11% non-Hispanic Black, 72% non-Hispanic White, and 5% multiracial or other race (Alaska Native or American Indian, Asian, or nonspecified). In the weighted population from 1998 to 2000, 46% of people with diabetes were 55 years and older, while 27% of people without diabetes were 55 years and older. In the weighted population from 2016 to 2018, 56% of people with diabetes were 55 years and older, while 38% of people without diabetes were 55 years and older. The average marginal effect of diabetes on probability of labor force participation was −10.9 percentage points (95% CI, −13.0 to −8.9) from 1998 to 2000 and −11.0 percentage points (95% CI, −13.0 to −9.1) from 2016 to 2018; for people who received Supplemental Security Income or Social Security Disability Insurance income, it was 4.4 percentage points (95% CI, 3.3-5.5) and 4.9 percentage points (95% CI, 3.7-6.0) from 1998 to 2000 and 2016 to 2018, respectively. During the same period, average marginal effects for all examined health outcomes significantly improved. Similar patterns were observed using BRFSS data, but with a slight improvement in labor force participation between 2017 to 2019 and 2021 to 2023.

**Conclusions and Relevance:**

This cross-sectional study demonstrated that while people with diabetes experienced meaningful health improvements, they saw little progress in economic performance. Changing patient selection appears to play a role. Future research is needed to disentangle the paradox.

## Key Points

**Question:**

Have the adverse economic consequences associated with diabetes mitigated over time?

**Findings:**

In this cross-sectional study including 249 712 respondents to the 1998 to 2018 National Health Interview Survey, individuals aged 40 to 64 years diagnosed with diabetes were 8 to 11 percentage points less likely to be in the labor force and 4 to 6 percentage points more likely to claim disability insurance income than observationally identical peers, with no significant change over time. Conversely, diabetes-associated health decrements significantly improved during the same period.

**Meaning:**

While people with diabetes experienced meaningful health improvements between 1998 and 2018, they did not experience progress in economic outcomes.

## Teaser

This cross-sectional study explores whether the health improvements of people with diabetes have matched the economic progress of this patient population.

## Introduction

The prevalence of diagnosed diabetes in the US has grown substantially during the 21st century and is projected to increase to 18% by 2060.^1,2^ People with diabetes face significant challenges, including higher mortality, devastating health complications (eg, limb amputation, myocardial infarction, end-stage kidney disease), and lost economic opportunities.^1,3,4,5,6,7^ Addressing these challenges is important for promoting societal well-being and productivity.

Advances in medical technology have improved diabetes detection and treatment, and the risks of diabetes-related complications and death decreased from the 1990s to the 2010s.^8,9,10,11^ Past research has found significant labor market penalties for people with diabetes—they are less likely to be in the labor market and employed, and they earn lower average wages than people without diabetes.^3,4,12,13^ However, little is known about whether these penalties have been reduced over time. Have advances in health outcomes been accompanied by concomitant improvements in labor market performance?

In this cross-sectional study, we examine long-run trends in labor force participation for people with diagnosed diabetes. Using repeated cross-sections of nationally representative data from 1998 to 2018, we estimate the gap in labor market participation associated with diabetes while accounting for observable characteristics and comorbidities.

## Methods

This cross-sectional study used data from the 1998 to 2018 National Health Interview Survey (NHIS), a nationally representative, annual survey on the health, behaviors, and health care of the noninstitutionalized US population. Major survey redesigns in 1997 and 2019 restricted our ability to make direct comparisons over time beyond our study period.^14^ We used harmonized NHIS data obtained from IPUMS.^15^ This study used deidentified publicly available data and therefore was not human participant research according to 45 CFR §46. We followed the Strengthening the Reporting of Observational Studies in Epidemiology (STROBE) reporting guideline.

The sample included nonpregnant adults aged 40 to 64 years. We focused on this age group because: (1) this period includes people’s highest earning years, before the normal retirement age, and (2) most diabetes diagnoses occur in persons aged between 45 and 64 years, with relatively few patients with prevalent diabetes among younger groups. A total of 263 586 potential observations met these criteria, and we dropped 13 874 with missing values for any analysis variables. We pooled observations into 3-year time periods to increase precision of estimates.

The main explanatory variable was a binary indicator of diagnosed diabetes, which was self-reported as ever being diagnosed by a physician or health care professional. We could not distinguish between type 1 and type 2 diabetes, so the diabetes indicator included both. eFigure 1 in Supplement 1 shows diagnosed diabetes prevalence during the study period.

The main outcomes were whether the respondent was in the labor force, defined as having a job or looking for one during the past week, and whether they received any income from Social Security Disability Insurance (SSDI) or Supplemental Security Income (SSI) in the past year. eFigure 2 in Supplement 1 shows labor force participation in our sample aligned with estimates from the Current Population Survey, which is used in official statistics.

To explore health mechanisms, we included the following self-reported outcomes: whether the respondent’s health limited any activities; whether the respondent's health limited their kind or amount of work (a subcategory of activities); whether the respondent spent any nights in hospital in the past year, as hospitalizations can cause lasting reductions in work and earnings^16^; and receipt of care from a health professional at least 10 times in the past year, as needing frequent health care visits could interfere with work. We also described trends in mortality, linked from the National Death Index, and whether people with diabetes report the condition limited any activities.

To address our limited ability to compare NHIS data before and after 2019, we conducted a supplemental analysis of Behavioral Risk Factor Surveillance System (BRFSS) data from 1993 to 2023. A drawback of these data is that the set of outcome variables available in all years was more limited; we focused on whether the respondent was ever employed in the past year and their self-rated health. BRFSS underwent a major sampling redesign in 2011, and trends before and after this year should be interpreted with caution.

### Statistical Analysis

We first described trends in average outcomes stratifying by diagnosed diabetes status. We fit trend lines using second-order fractional polynomials.^17^ We summarized socioeconomic characteristics and health risks by diabetes status and described differences in 2 periods (1998-2000 and 2016-2018). In each period, we compared characteristics between people with and without diabetes, with statistical significance established based on the *F* statistic resulting from a Rao-Scott second-order correction to the Pearson χ^2^ test to account for the complex sample design.^18^ To evaluate temporal changes, we used survey-adjusted logit regressions with fully interacted diabetes and time indicators to estimate the difference-in-differences and evaluated significance using a Wald *z* test.

We then estimated outcome differences associated with diabetes after adjusting for other observable characteristics. We estimated associations between diabetes and the outcomes using probit regressions, with diabetes and all controls fully interacted with time period dummies, and computed the average marginal effect^19^ of diabetes in each period. We sequentially added controls for (1) age, sex, self-reported race and ethnicity (Hispanic, non-Hispanic Black, non-Hispanic White, and non-Hispanic other race [Alaska Native or American Indian, Asian, multiracial, or nonspecified]), marital status, census region, and nativity; (2) educational attainment; and (3) body mass index, smoking (at least 100 cigarettes in respondent’s lifetime), and ever diagnosed with hypertension, lung disease (emphysema, asthma, or chronic bronchitis), and cancer.

All analyses used survey weights provided by NHIS to generate nationally representative estimates. We used a 2-tailed α < .05 as the critical value to determine statistical significance. Statistical tests and 95% CIs used NHIS’s stratum and primary sampling unit variables to account for the complex sample design. Analyses were conducted from September 2023 to November 2024 using Stata software, version 18 (StataCorp LLC).

## Results

The study sample was composed of 249 712 respondents, including 25 177 with diabetes (eTable in Supplement 1). The weighted population was 50% female, 12% Hispanic, 11% non-Hispanic Black, 72% non-Hispanic White, and 5% multiracial or other race (Alaska Native or American Indian, Asian, or nonspecified). People with diabetes were older, more likely to be male, less likely to be non-Hispanic White, had lower education levels, were more likely to smoke, had higher body mass index, and had higher rates of other chronic conditions. In the weighted population from 1998 to 2000, 46% of people with diabetes were 55 years and older, while 27% of people without diabetes were 55 years and older. In the weighted population from 2016 to 2018, 56% of people with diabetes were 55 years and older, while 38% of people without diabetes were 55 years and older.

**Figure 1.**
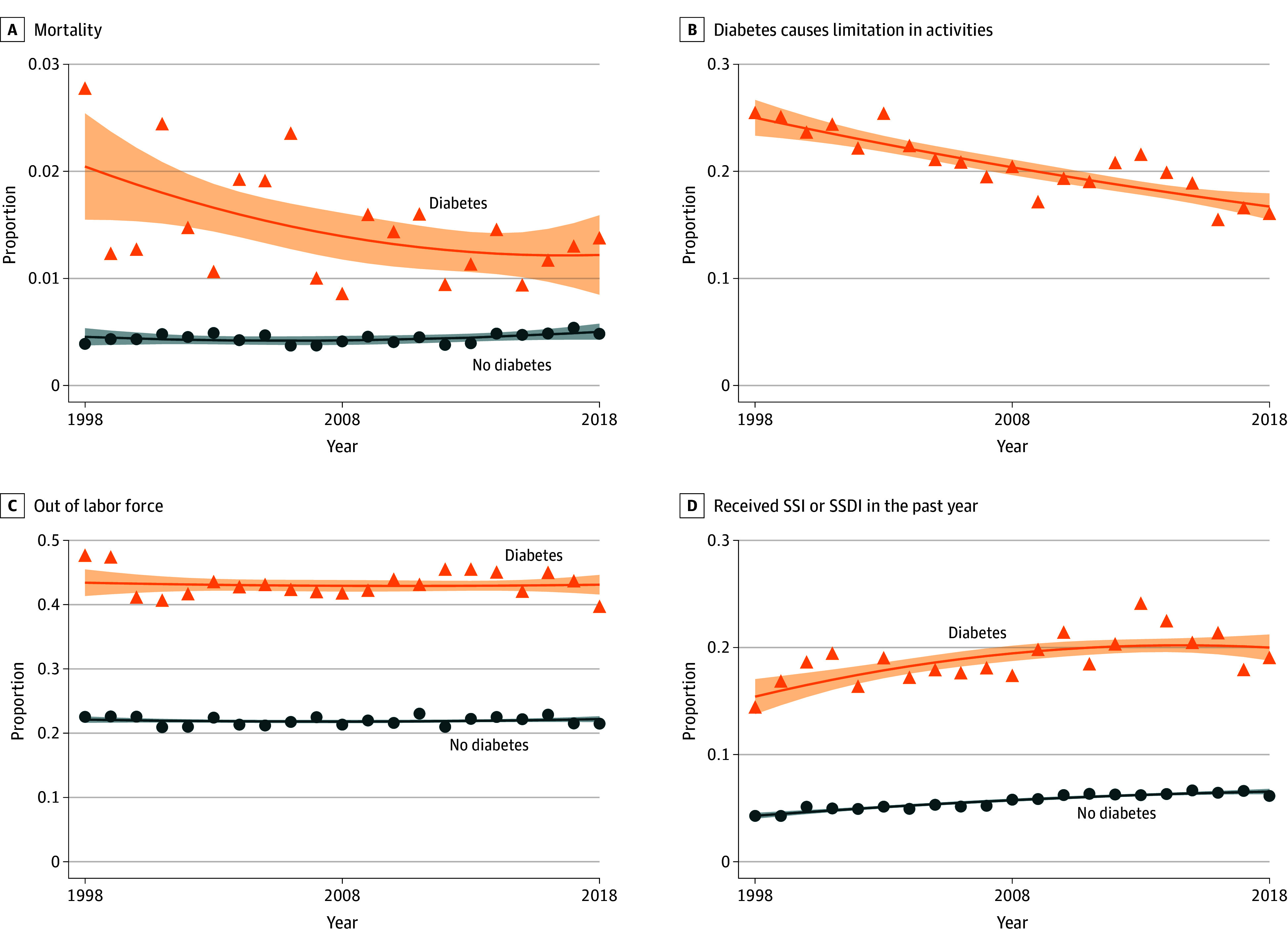
Trends in Health and Economic Outcomes by Diagnosed Diabetes Status, Individuals in the US Aged 40 to 64 Years This figure shows the proportion of the population with the given outcome in each year, stratified by diagnosed diabetes status. Orange triangles indicate the proportion among the population with diagnosed diabetes, and blue circles indicate the proportion among people without diabetes. Fitted lines are second-order fractional polynomials with 95% CIs presented as the shaded areas. National Health Interview Survey (NHIS) sample adult weights were used to generate nationally representative estimates; mortality used weights adjusted for linkage to the National Death Index. Data were sourced from the NHIS, 1998 to 2018, obtained from IPUMS. SSDI indicates Social Security Disability Insurance; SSI, Supplemental Security Income.

Trends in health outcomes showed relative improvement over time: people with diabetes became less likely to report that their condition caused limitations in activities, and they had decreasing rates of mortality (Figure 1). In contrast, there was not a similar convergence in labor market outcomes: an approximately 20–percentage point labor force participation gap remained nearly constant over time. Similarly, people with diabetes persistently exhibited SSI/SSDI claiming rates 3 times higher than those without diabetes.

The Table shows that the populations with and without diabetes differed along many characteristics, and these differences changed over time. In particular, the proportion of the overall population with a Bachelor’s degree rose over time, but it rose more slowly for the diabetes population, and diabetes prevalence increased more quickly among lower-educated groups (eFigure 1 in Supplement 1). As a result, people with diabetes were 16 percentage points less likely to have a Bachelor’s degree than those without diabetes from 2016 to 2018, a 6–percentage point larger difference than from 1998 to 2000.

**Table.** Characteristics of the Population Aged 40 to 64 Years With and Without Diabetes by Time Period

Characteristic	Proportion^a^					
	1998-2000		2016-2018		Difference in differences^b^	
	No diabetes	Diabetes	No diabetes	Diabetes	Estimate	*P* value
Demographics						
Age >55 y	0.27	0.46	0.38	0.56	−0.01	.61
Sex						
Female	0.50	0.48	0.51	0.47	−0.01	.37
Male	0.50	0.52	0.49	0.53	0.01	
Race and ethnicity						
Hispanic	0.08	0.13	0.14	0.20	0.00	.01
Non-Hispanic Black	0.10	0.19	0.11	0.16	−0.04	
Non-Hispanic White	0.78	0.64	0.67	0.57	0.04	
Other	0.04	0.05	0.08	0.08	−0.01	
Married	0.71	0.68	0.66	0.58	−0.05	.001
>Bachelor's degree	0.27	0.17	0.38	0.22	−0.06	<.001
Born in the US	0.88	0.87	0.79	0.80	0.02	.07
Labor force participation, male	0.85	0.61	0.84	0.63	0.02	.27
Labor force participation, female	0.70	0.48	0.72	0.51	0.01	.63
SSI or SSDI^c^	0.05	0.17	0.06	0.19	0.01	.45
Health						
Ever smoker	0.53	0.57	0.39	0.47	0.04	.03
Obese (BMI >30)	0.23	0.51	0.32	0.61	0.01	.45
Obese category 3 (BMI >40)	0.02	0.10	0.05	0.15	0.03	.002
High blood pressure	0.25	0.61	0.31	0.69	0.02	.14
Heart disease	0.10	0.28	0.09	0.23	−0.05	.001
Lung disease^d^	0.12	0.19	0.15	0.23	0.02	.24
Cancer	0.06	0.07	0.09	0.09	0.00	.89
Health limits any activities	0.14	0.41	0.14	0.36	−0.06	<.001
Health limits work	0.11	0.35	0.12	0.32	−0.04	.003
Any nights in the hospital	0.07	0.21	0.06	0.16	−0.04	<.001
>10 Annual health care visits	0.11	0.34	0.12	0.28	−0.06	<.001
Observations, No.	33 240	2598	29 407	3761	NA	NA

Abbreviations: BMI, body mass index (calculated as weight in kilograms divided by height in meters squared); NA, not applicable; SSDI, Social Security Disability Insurance; SSI, Supplemental Security Income.

^a^ National Health Interview Survey sample adult weights were used to generate nationally representative estimates. All differences between the diabetes and no diabetes populations are statistically significant at the *P* < .05 level, except for being born in the US and cancer, based on the *F* statistic obtained from a Rao-Scott second-order correction to the Pearson χ^2^ to account for the National Health Interview Survey (NHIS) complex sample design. The data were sourced from the NHIS from 1998 to 2000 and the NHIS from 2016 to 2018, obtained from IPUMS.

^b^ Difference-in-differences column shows the difference over time (2016-2018 minus 1998-2000) in the differences between the diabetes and no diabetes populations. Differences were estimated using survey design–corrected regressions of the characteristic on fully interacted diabetes and time period indicators, using logit regression for binary characteristics and multinomial logit regressions for categorical characteristics. *P* values were calculated using adjusted Wald *z* tests.

^c^ SSI or SSDI refers to this type of income receipt in the past year.

^d^ Lung disease includes emphysema, asthma, or chronic bronchitis.

**Figure 2.**
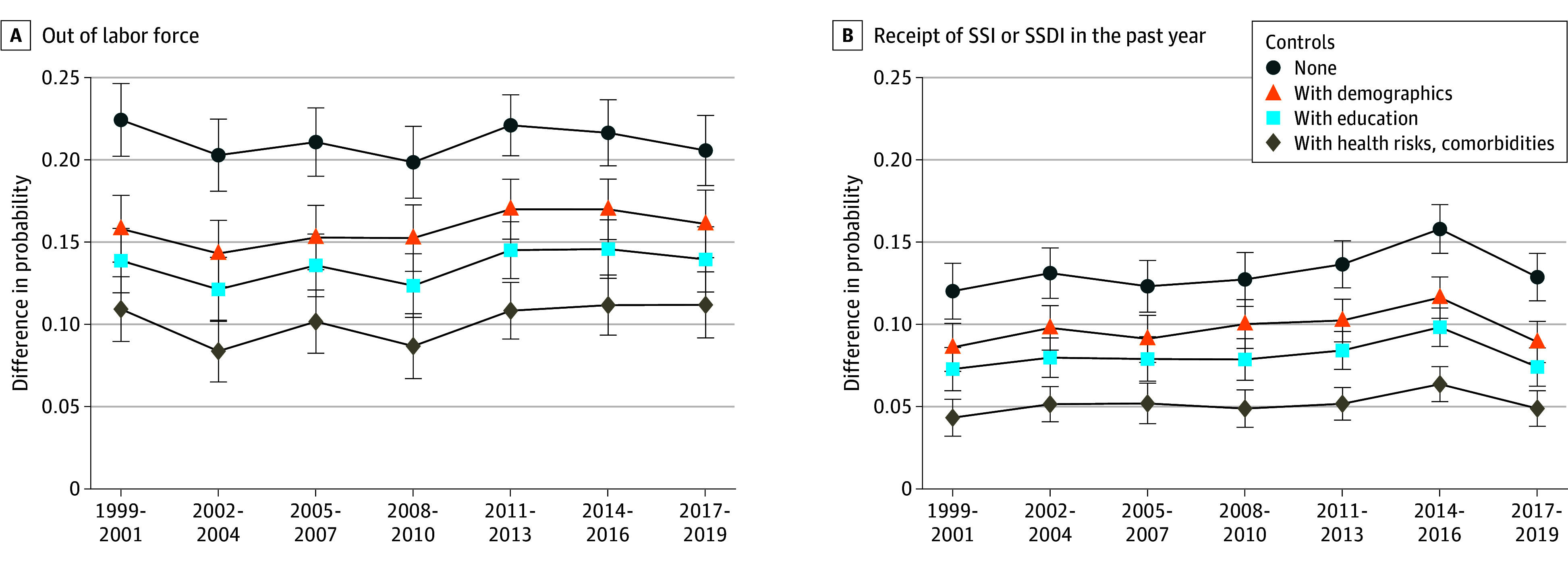
Regression-Adjusted Differences in Economic Outcomes Among Individuals in the US Aged 40 to 64 Years With Diabetes Relative to Without Diabetes This figure shows point estimates with 95% CIs (error bars) for the average marginal effect of diabetes within each time period. Average marginal effects are calculated based on survey-adjusted probit regressions with diabetes and included controls fully interacted with time period dummies. Estimates used National Health Interview Survey (NHIS) sample adult weights, and 95% CIs were adjusted for the complex sample design. Demographics include age (5-year categories), sex, race and ethnicity (Hispanic, non-Hispanic Black, non-Hispanic White, and non-Hispanic other race [Alaska Native or American Indian, Asian, multiracial, or nonspecified]), marital status interacted with sex, census region, whether the respondent was born in the US, and education (less than high school, high school, some college, and Bachelor’s degree or more). Health risks include body mass index (5 categories) and whether the respondent has smoked at least 100 cigarettes in their life, and comorbidities include ever being diagnosed with hypertension, lung disease (asthma, emphysema, or chronic bronchitis), and cancer. The year 2020 is excluded because disruptions due to the onset of the COVID-19 pandemic might have impacted data collection and quality, and labor force outcomes might have been temporarily impacted due to circumstances unrelated to the long-term trends of interest for this study. Data were sourced from the NHIS, 1998 to 2018, obtained from IPUMS. SSDI indicates Social Security Disability Insurance; SSI, Supplemental Security Income.

Figure 2 reports regression-adjusted differences in labor force participation and SSI/SSDI over time, sequentially adding controls for observable characteristics. Diabetes was associated with a 20.2–percentage point (95% CI, 18.1-22.5) to 22.4–percentage point (95% CI, 20.2-24.6) higher probability of being out of the labor force in the unadjusted model. Differences in demographics and education accounted for 33.4% to 40.1% of this difference. After adding controls for health risks and comorbidities, an 8.4–percentage point (95% CI, 6.5-10.2) to 11.2–percentage point (95% CI, 9.2-13.2) gap in labor force participation remained, with no evidence of improvement over time. Similar patterns were observed for disability claiming: holding observable characteristics constant, people with diabetes were 4.3 percentage points (95% CI, 3.2-5.4) to 6.4 percentage points (95% CI, 5.3-7.4) more likely to be claiming SSI/SSDI, with little change over time. eFigure 3 in Supplement 1 shows results are robust to including mental health controls.

**Figure 3.**
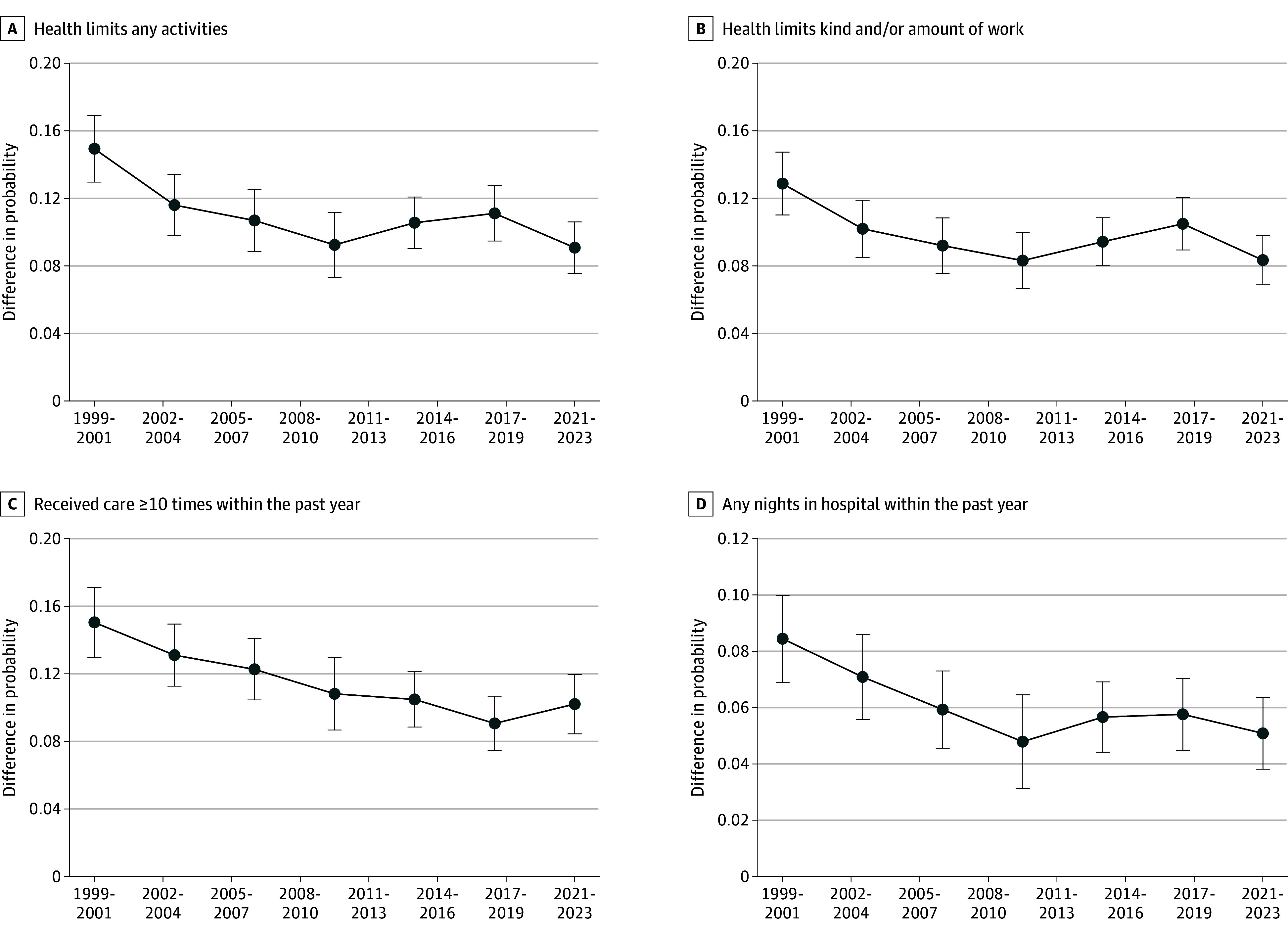
Regression-Adjusted Differences in Health Outcomes Among Individuals in the US Aged 40 to 64 Years With Diabetes Relative to Without Diabetes This figure shows point estimates with 95% CIs (error bars) for the average marginal effect of diabetes within each time period. Average marginal effects are calculated based on survey-adjusted probit regressions with diabetes and all controls described in Figure 2 notes fully interacted with time period dummies. Estimates used National Health Interview Survey (NHIS) sample adult weights, and 95% CIs were adjusted for the complex sample design. The year 2020 is excluded because disruptions due to the onset of the COVID-19 pandemic might have impacted data collection and quality, and labor force outcomes might have been temporarily impacted due to circumstances unrelated to the long-term trends of interest for this study. Data were sourced from the NHIS, 1998 to 2018, obtained from IPUMS.

The adjusted difference in the probability of hospitalization and frequently needing health care associated with diabetes declined by nearly one-third over time (Figure 3). Holding observable characteristics fixed, diabetes was associated with a 8.5–percentage point (95% CI, 6.9-10.0) higher probability of any hospitalization from 1998 to 2000, which declined to a 5.1–percentage point (95% CI, 3.8-6.4) difference by 2016 to 2018; the association with frequently needing care similarly declined from a 15.0 (95% CI, 13.0-17.1) percentage point difference to 10.2–percentage point (95% CI, 8.4-12.0). The associated difference in reporting health limits any activities also declined by approximately one-third, from 14.9 (95% CI, 13.0-16.9) down to a difference of 9.1 (95% CI, 7.6-10.6) percentage points. Adding to the paradox, the results showed improvements in respondents’ reports that their health limits work.

**Figure 4.**
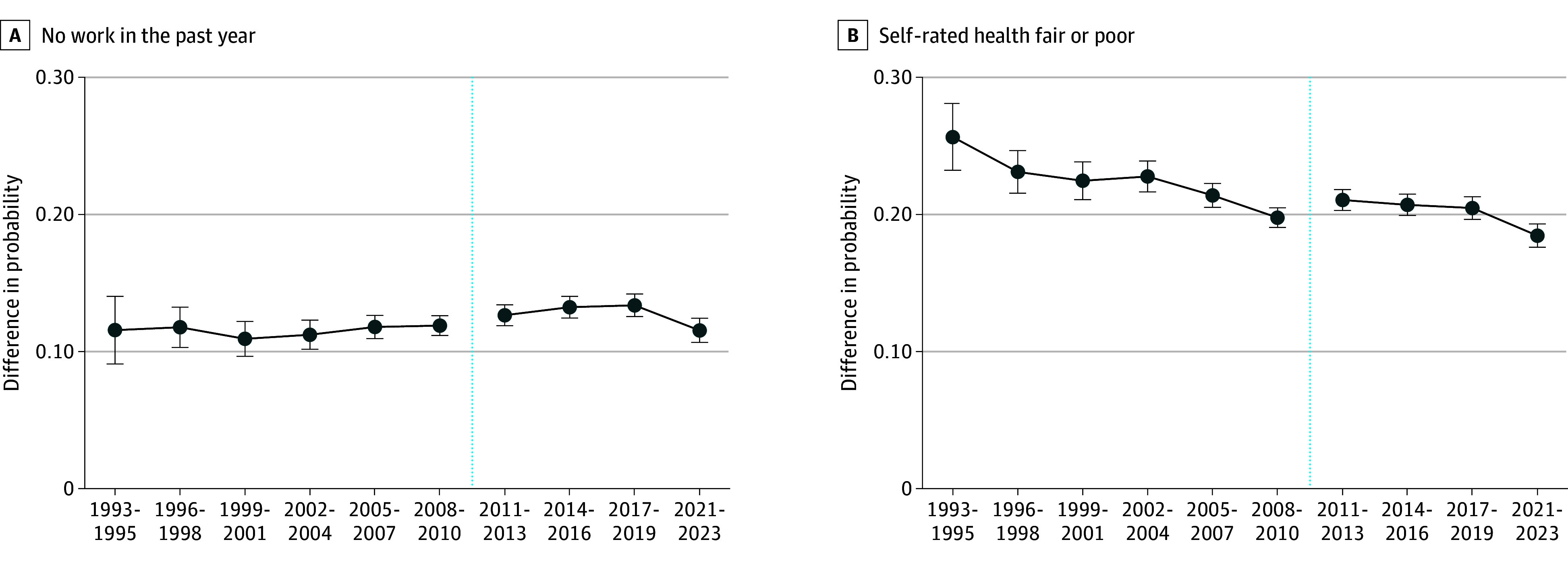
Regression-Adjusted Differences in Outcomes Using Alternative Dataset Among Individuals in the US Aged 40 to 64 Years With Diabetes Relative to Without Diabetes This figure shows point estimates with 95% CIs (error bars) for the average marginal effect of diabetes within each time period. Average marginal effects are calculated based on survey-adjusted probit regressions with diabetes and all controls fully interacted with time period dummies. Controls included age, sex, race and ethnicity (Hispanic, non-Hispanic Black, non-Hispanic White, and non-Hispanic other race [Alaska Native or American Indian, Asian, multiracial, or nonspecified]), marital status, education, body mass index, whether the respondent has smoked at least 100 cigarettes, and state dummies. Estimates used sample weights. The year 2020 is excluded because disruptions due to the onset of the COVID-19 pandemic might have impacted data collection and quality, and labor force outcomes might have been temporarily impacted due to circumstances unrelated to the long-term trends of interest for this study. The vertical dotted lines indicate the change in sample and weighting design. Data were sourced from the Behavioral Risk Factor Surveillance System, 1993 to 2023.

Finally, to check the robustness of these estimates to other data contexts and to extend the time period of study, Figure 4 presents estimates of the marginal effect of diabetes on employment and self-rated health in BRFSS. Again, the average marginal effect of diabetes on the probability of reporting that health is fair or poor significantly decreased. Conversely, the average marginal effect on the probability of no work in the past year slightly increased over most of the same time period. However, a slight reduction in this negative employment association in the last time period was observed in the study sample. Still, this recent improvement did not make up for the long-term trend, and the marginal difference in employment for people with diabetes remained no better from 2021 to 2023 than it was in the 1990s and early 2000s.

## Discussion

Using nationally representative data to perform a cross-sectional analysis, we described trends in the health and economic performance of people with diabetes since the 1990s. We found that people with diabetes have experienced significant progress in health outcomes, with the bulk of the gains occurring from the late 1990s to 2010. But throughout the 2-decade period, we observed little economic progress as measured by labor force participation and disability claiming.

The lack of economic convergence is concerning given the large increase in diabetes prevalence, which implies an increasing total labor market burden. In addition to reducing productivity, this may have increased strain on government budgets if people with diabetes are more likely to use disability insurance (DI) programs, as we found. This aligns with findings from the American Diabetes Association’s estimates of the total macroeconomic burden of diabetes in the US; they found the total economic cost (including direct medical cost and indirect costs from premature mortality and lost productivity) increased with each 5-year estimate between 2007 and 2022.^5,20^

The lack of economic progress is puzzling given the improvements in health outcomes among people with diabetes. Why have health improvements not translated to improving economic performance? One potential explanation relates to changes in patient selection. We documented that the composition of the diabetes population has changed over time along a number of observable characteristics as prevalence has increased. In particular, differences in education have widened such that the average educational attainment among people with diabetes compared to the total population is relatively lower in more recent years. As there have also been important changes over time in the value of education in the labor market,^21,22^ we would expect the increasingly negative selection on education to contribute to a relative reduction in average economic performance.

We adjust for important observable characteristics, but unobserved factors not directly included in our models could also be at play. For example, the average effect of educational attainment levels might not fully capture differences in education, cognitive ability, or other important skills. Gensowski and Gørtz^23^ found that a large proportion of the education-health gradient is explained by differences in socio-emotional skills. Such skills may also be important for labor market outcomes; however, we cannot observe these dimensions in our data, and they could lead to omitted variable bias.

We posit that the changing composition of the diagnosed diabetes population is related to technological and policy improvements with impacts on 3 important margins: prevention, diagnosis, and mortality.

First, advances in monitoring and identification of prediabetes^24,25,26^ improve people’s ability to have an early warning of their diabetes risks, and those with more resources or ability to reverse course may be less likely to progress to diagnosed diabetes status. If characteristics that are associated with one’s ability to prevent diabetes onset also correlate with economic potential, then increasingly the individuals who develop diabetes might have fewer economic prospects independent of their disease status. Goldman and Smith^27,28^ show there are socioeconomic differences in the adoption of medical technologies and self-management of health conditions. A large education gradient in health outcomes expanded over time and could be due to differences in prevention and self-management abilities,^29^ for which education categories may be a rough proxy. We similarly found that an education gradient in diagnosed diabetes prevalence continued to widen since their study ended in 2006.

Second, socioeconomic status (SES) and other unobserved characteristics can influence the probability that someone with diabetes is aware of their condition and diagnosed. Smith^30^ found that education and income gradients in the proportion of diabetes diagnoses rose from 1970 to 2000. By 2000, individuals with higher SES were more likely to be diagnosed. Since then, screening efforts and criteria evolved,^31,32,33^ and more people became diagnosed who might have otherwise remained unaware of their emerging diabetes,^34,35,36^ which likely continued to alter the relationship between individual characteristics and probability of diagnosis. Wang and coauthors^36^ found no education gradient in the proportion of diabetes diagnosed from 2017 to 2020, suggesting the gradient Smith^30^ found had flattened. Fang and colleagues^34^ found some differences in SES from 1988 to 2010 and 2011 to 2018, but conclusions about the gradient and changes over time differ depending on the definition of undiagnosed and the measure of SES (income or education). New diabetes diagnoses among Medicaid enrollees surged following the eligibility expansions that occurred in the 2010s,^37,38^ suggesting an increase in newly diagnosed people with low income. These changes have implications for the composition of the diagnosed population. For example, if improvements in screening and access disproportionately increase identification of diabetes among people with lower SES while also identifying it at earlier stages before sequelae emerge, the diagnosed population might shift to look healthier but with lower SES on average.

Finally, reductions in mortality probability for people with diabetes alter the composition of the diagnosed population because individuals who would have died are surviving longer. Combined with population aging^39^ and a trend toward earlier age at diagnosis,^40^ the duration of time that the average diagnosed person has spent living with diabetes likely increased during our study period. Consistent with this, the incident rate has been stable or slightly declining since 2000, even as prevalence grew.^1^

Another underlying factor that could be influencing the patterns we observed is the changing nature of work, which impacts labor force participation and DI claiming decisions. Butrica and Mudrazja^41^ found that job requirements are an important determinant of DI application independent of health and other characteristics, and the relationship has changed over time: associations between negative health and DI applications are amplified by physical requirements, and the jobs of DI applicants have become more physically demanding. It might be that the diagnosed diabetes population grew over time to include relatively more individuals likely to have physically demanding occupations (eg, men with lower education levels), and the changing nature of jobs amplified the relationship between diabetes and dropping out of the labor force to claim DI. Bloom et al^42^ found that the rise in work from home following the COVID-19 pandemic has further changed the disability and employment relationship, which disproportionately improved employment for people with disabilities. Using BRFSS data, we also found a small improvement in employment for people with diabetes in the post–COVID-19 pandemic period, potentially pointing to new opportunities for people with diabetes to engage in work in the future.

The dynamic selection explanation could help elucidate the drivers of plateaus in diabetes management,^43^ limited behavior change among people newly diagnosed with diabetes,^44^ and a resurgence in some diabetes complications^45^ emerging in the 2010s. If the composition of the population shifted to include relatively more people with challenges managing their health, this would portend downstream changes in diabetes-related complications and other adverse events. Gregg and coauthors^45^ also point to changing patient composition as one potential reason for resurging rates of complications.

Disentangling the dynamic, interacting forces we have discussed is important for informing strategies that can effectively reduce the economic disparities that people with diabetes face. Future research using panel data with more objective measures of diabetes status (eg, measured hemoglobin A_1c_) could be helpful for understanding who is most likely to transition into or out of diabetes risk states and how these transitions relate to the evolution of their labor market outcomes. Clinical trials for diabetes prevention and management therapies could incorporate more economic information into their data collection; the socioeconomic representativeness of trials is important for generalizing results to other contexts, and adding more focus on economic end points (eg, employment) could help identify strategies that improve health in ways that translate to productivity gains.

### Limitations

This study relied on self-reported survey data, which may be subject to misreporting or recall biases. The survey data provided self-reported information about whether respondents had ever been diagnosed with diabetes but did not include objective measures of underlying diabetes status (eg, measured hemoglobin A_1C_), which limits the ability to distinguish the extent to which trends have been influenced by changes in diagnosis patterns rather than disease incidence, as discussed previously. Moreover, the surveys did not distinguish between people with type 1 versus type 2 diabetes, and these individuals might have experienced different trends in health or economic outcomes. Finally, although the study controlled for differences over time in observed characteristics between individuals with and without diabetes, unobserved factors that differentially influenced trends in health or economic outcomes may be present, as discussed previously.

## Conclusions

This cross-sectional study documented a paradox: over the last 3 decades in the US, people with diagnosed diabetes have experienced substantial health gains, but associated economic decrements have remained persistently large. Understanding the diabetes paradox has important implications for emerging health trends as the nation’s population ages and for the long-run impacts of recent efforts to expand access to antiobesity medications, such as semaglutide.^46^ We posit that changing patient selection plays a role in producing these paradoxical trends. The social and economic contexts in which many people with diabetes live, in addition to the direct health impacts of the disease, are important to address to develop effective strategies that will reduce the labor market penalties associated with diabetes.

## Notes

Supplement 1. eFigure 1. Diagnosed diabetes prevalence over time by sex and education, Americans aged 40-64 years eFigure 2. Comparison of labor force participation in the National Health Interview Survey and the Current Population Survey, Americans aged 40-64 years eTable. Characteristics of sample adults aged 40-64 years, overall and by diagnosed diabetes status eFigure 3. Regression-adjusted differences in economic outcomes with controls for mental health, diabetes relative to no diabetes, Americans aged 40-64 years

Supplement 2. Data Sharing Statement
