# Supplementary material for: Long-Term Health Improvements and Economic Performance Among Individuals With Diabetes
Source: JAMA Health Forum. 2025 May 16;6(5):e250756. doi: 10.1001/jamahealthforum.2025.0756 (PMC12084845; doi:10.1001/jamahealthforum.2025.0756)
Supplement: Supplement 1. — eFigure 1. Diagnosed diabetes prevalence over time by sex and education, Americans aged 40-64 years eFigure 2. Comparison of labor force participation in the National Health Interview Survey and the Current Population Survey, Americans aged 40-64 years eTable. Characteristics of sample adults aged 40-64 years, overall and by diagnosed diabetes status eFigure 3. Regression-adjusted differences in economic outcomes with controls for mental health, diabetes relative to no diabetes, Americans aged 40-64 years [file jamahealthforum-e250756-s001.pdf]

Supplemental Online Content

Chapel JM, Goldman DP, Kahn ME, Tysinger B. Long-term health improvements and economic performance among individuals with diabetes. *JAMA Health Forum.* 2025;6(5):e250756. doi:10.1001/jamahealthforum.2025.0756

eFigure 1. Diagnosed diabetes prevalence over time by sex and education, Americans aged 40-64 years

eFigure 2. Comparison of labor force participation in the National Health Interview Survey and the Current Population Survey, Americans aged 40-64 years

eTable. Characteristics of sample adults aged 40-64 years, overall and by diagnosed diabetes status

eFigure 3. Regression-adjusted differences in economic outcomes with controls for mental health, diabetes relative to no diabetes, Americans aged 40-64 years

This supplemental material has been provided by the authors to give readers additional information about their work.

eFigure 1. Diagnosed diabetes prevalence over time by sex and education, Americans aged 40-64 Years

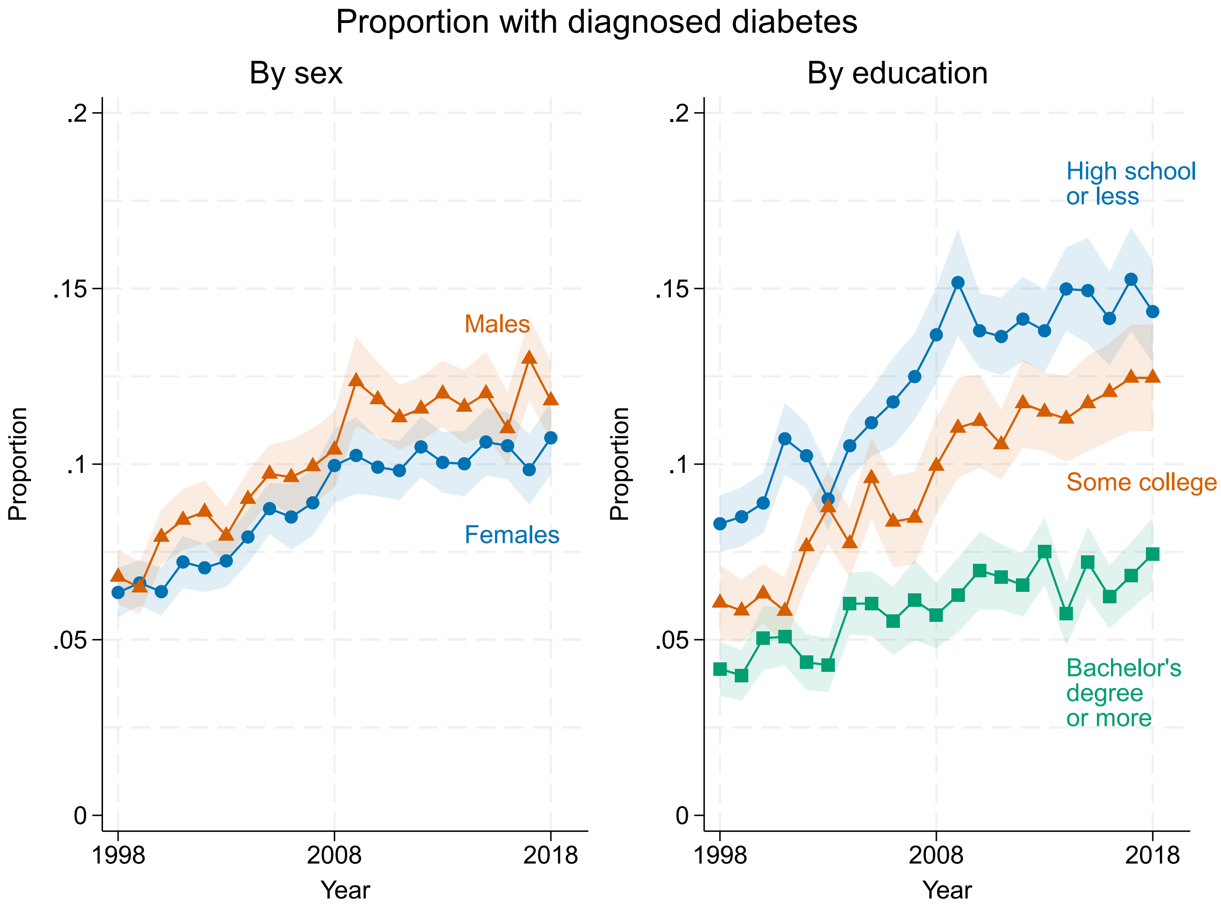

Source: National Health Interview Survey (NHIS), 1998–2018, obtained from IPUMS.
Notes: This figure shows the annual prevalence of diagnosed diabetes among the study sample described in the text. The left panel shows prevalence stratified by sex; blue circles indicate the proportion with diagnosed diabetes among females and orange triangles indicate the proportion among males. The right panel shows prevalence stratified by educational attainment; blue circles indicate the proportion with diagnosed diabetes among respondents with a high school degree (including GED) or less, orange triangles indicate the proportion among respondents with some college (including Associate's degree or some years of college but no degree), and green squares indicate the proportion among respondents with a Bachelor's degree or more. NHIS sample adult weights were used to generate nationally representative estimates. 95% confidence intervals were adjusted to account for the NHIS complex sample design.

© 2025 Chapel JM et al. *JAMA Health Forum*.

eFigure 2. Comparison of labor force participation in the National Health Interview Survey and the Current Population Survey, Americans aged 40-64 Years

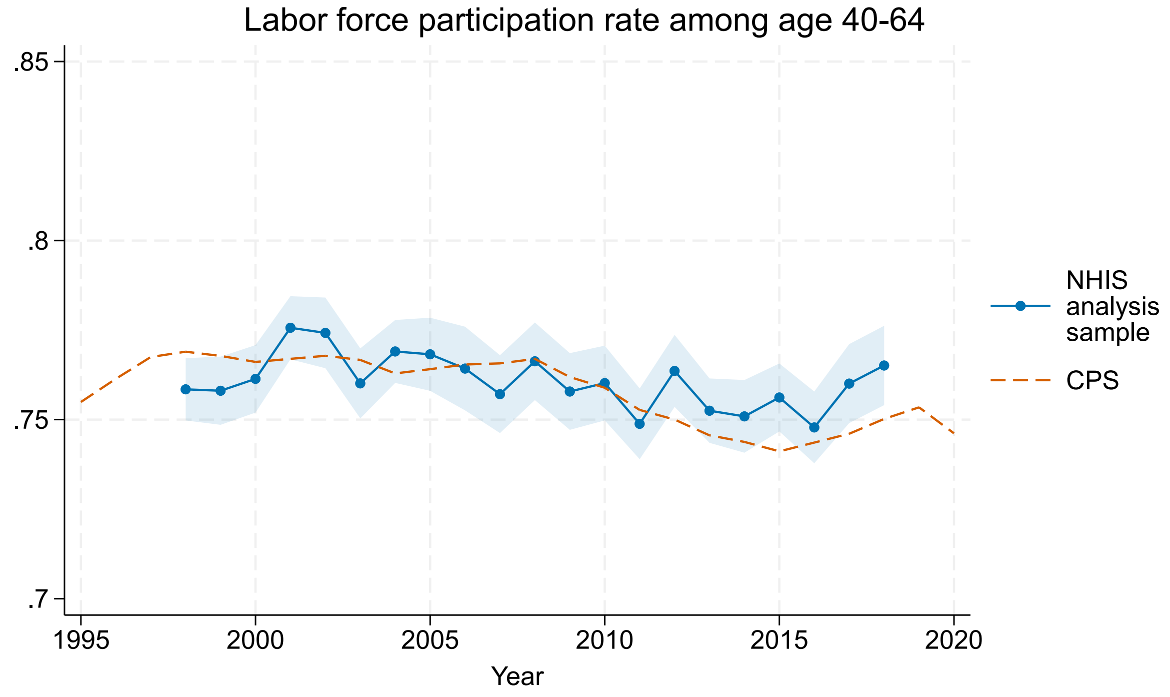

Sources: National Health Interview Survey (NHIS), 1998–2018, and the monthly Current Population Survey (CPS), 1995–2020, obtained from IPUMS.

Notes: This figure shows the annual labor force participation rate among the study sample described in the text and among monthly CPS respondents from the same age group for comparison. Labor force participation was defined as being employed, on layoff, or looking for work in the past week. Monthly CPS respondents are pooled by year. NHIS sample adult weights and CPS person-level final basic weights were used to generate nationally representative estimates. 95% confidence intervals around NHIS point estimates were adjusted to account for the NHIS complex sample design.

© 2025 Chapel JM et al. *JAMA Health Forum*.

eTable. Characteristics of sample adults aged 40-64 Years, overall and by diagnosed diabetes status

	All (n = 249,712)	No diabetes (n = 224,535)	Diabetes (n = 25,177)	*P*-value
**Demographics**				
Age				
40-44	0.22	0.24	0.11	<0.001
45-49	0.22	0.23	0.15	
50-54	0.21	0.21	0.21	
55-59	0.18	0.18	0.25	
60-44	0.16	0.15	0.27	
Sex[a]				
Female	0.50	0.51	0.47	<0.001
Male	0.50	0.49	0.53	
Race/ethnicity				
Hispanic	0.12	0.11	0.16	<0.001
Non-Hispanic Black	0.11	0.11	0.17	
Non-Hispanic White	0.72	0.73	0.61	
Other[b]	0.05	0.05	0.06	
Born in the US	0.84	0.84	0.83	0.001
Marital status				
Married	0.67	0.68	0.62	<0.001
Divorced/separated	0.19	0.19	0.21	
Widowed	0.03	0.03	0.05	
Never married	0.11	0.11	0.12	
Educational attainment				
Less than high school	0.13	0.12	0.21	<0.001
High school, GED	0.28	0.27	0.31	
Some college	0.29	0.29	0.29	
Bachelor's degree or more	0.31	0.32	0.19	
Labor force participation, females	0.70	0.72	0.50	<0.001
Labor force participation, males	0.82	0.84	0.63	<0.001
SSI or SSDI income[c]	0.07	0.06	0.19	<0.001
**Health**				
Obesity category (BMI)[d]				
Normal weight (18.5-24	0.31	0.33	0.12	<0.001
Overweight (25-29)	0.37	0.38	0.29	
Obese I (30-34)	0.19	0.18	0.28	
Obese II (35-39)	0.07	0.06	0.16	
Obese III (40+)	0.05	0.04	0.15	
Underweight (<18.5)	0.01	0.01	0.00	
Ever smoker	0.46	0.45	0.50	<0.001
High blood pressure	0.33	0.29	0.68	<0.001
Heart disease	0.11	0.10	0.26	<0.001
Lung disease[e]	0.14	0.14	0.22	<0.001
Cancer	0.08	0.07	0.10	<0.001
Health limits any activities	0.16	0.14	0.38	<0.001
Health limits work	0.14	0.12	0.33	<0.001
Any nights in hospital	0.08	0.07	0.18	<0.001

© 2025 Chapel JM et al. *JAMA Health Forum*.

Received healthcare 10+ times	0.13	0.12	0.29	<0.001

Source: National Health Interview Survey (NHIS), 1998–2018, obtained from IPUMS.
Notes: This table summarizes the characteristics of the analysis sample overall and stratified by diabetes status. Each cell shows the proportion of the population (specified in columns) with the given characteristic (specified in rows). NHIS sample adult weights were used to generate nationally representative estimates. *P*-values from the *F*-statistic obtained from a Rao-Scott second order correction to the Pearson $\chi 2$ test to account for the NHIS complex sample design.
a. Sex was self-reported as a binary choice between male or female.
b. Other includes Alaskan Native or American Indian, Asian, multiple races, or non-specified.
c. SSI or SSDI refers to Supplemental Security Income or Social Security Disability Insurance income receipt in the past year.
d. BMI, body mass index [weight (kg)/height (m)2].
e. Lung disease includes emphysema, asthma, or chronic bronchitis.

© 2025 Chapel JM et al. *JAMA Health Forum*.

eFigure 3. Regression-adjusted differences in economic outcomes with controls for mental health, diabetes relative to no diabetes, Americans aged 40-64 Years

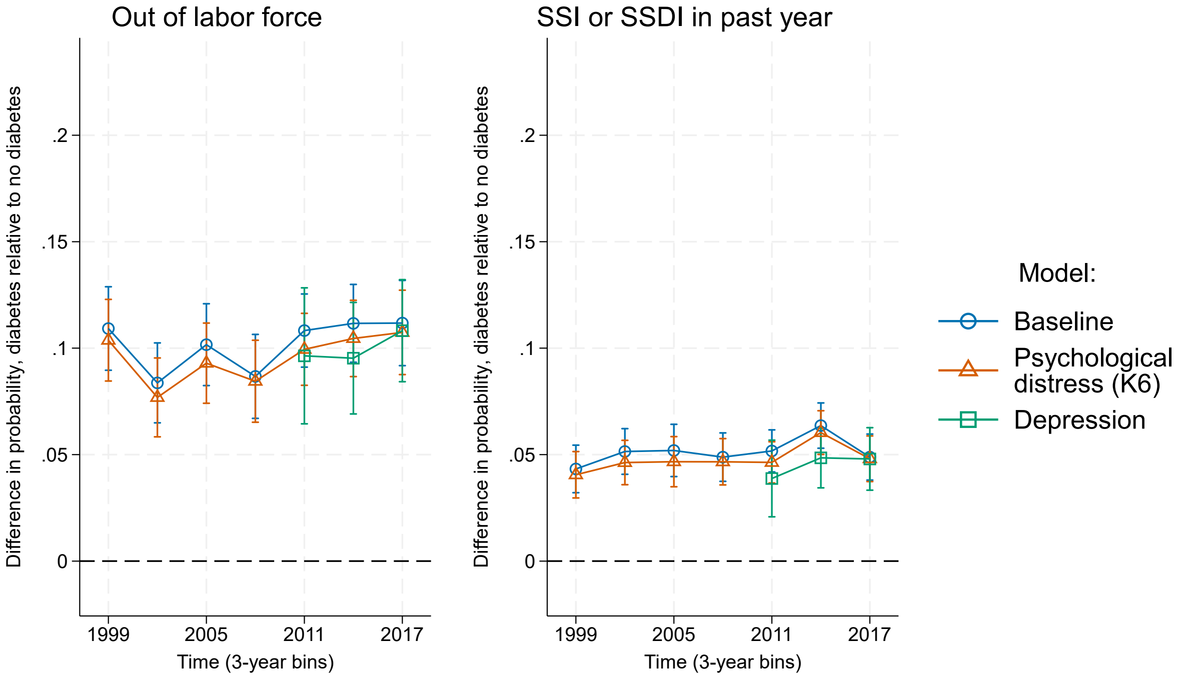

Source: National Health Interview Survey (NHIS), 1998–2018, obtained from IPUMS.
Notes: This figure shows point estimates with 95% confidence intervals for the average marginal effect of diabetes within each time period. Average marginal effects are calculated based on survey-adjusted probit regressions with diabetes and included controls fully interacted with time period dummies. Estimates used NHIS sample adult weights and 95% confidence intervals were adjusted for the complex sample design. SSI or SSDI refers to Supplemental Security Income or Social Security Disability Insurance income receipt in the past year. The baseline model includes all controls: age (5-year categories), sex, race/ethnicity (Hispanic, non-Hispanic Black, non-Hispanic White, and non-Hispanic other [Alaskan Native or American Indian, Asian, multiple races, or non-specified]), marital status interacted with sex, census region, whether the respondent was born in the US, education (less than high school, high school, some college, and Bachelor’s degree or more), BMI (5 categories), whether the respondent has smoked 100+ cigarettes in their life, and whether ever diagnosed with hypertension, lung disease (asthma, emphysema, or chronic bronchitis), and cancer. The psychological distress (K6) model adds a binary control indicating severe mental illness according to the battery of questions used in the Kessler 6 scale; we summed the six component questions (each range from 0 to 4) and used a score of 13 or higher to indicate severe mental illness, following the criteria proposed by Kessler. The depression model instead adds a control based on the questions “How often do you feel depressed?” and “Do you take prescription medication for depression?” These questions were included starting in 2010 and were only asked of a one-quarter subsample of adult respondents included in a quality of life supplement; they were asked of all sample adults starting in 2018. We defined depression as a binary variable indicating the respondent reported feeling depressed at least weekly or reported taking medication for depression.

© 2025 Chapel JM et al. *JAMA Health Forum*.
