# Supplementary material for: Long-Term Health Improvements and Economic Performance Among Individuals With Diabetes
Source: JAMA Health Forum. 2025 May 16;6(5):e250756. doi: 10.1001/jamahealthforum.2025.0756 (PMC12084845; doi:10.1001/jamahealthforum.2025.0756)
Supplement: Supplement 2. — Data Sharing Statement [file jamahealthforum-e250756-s002.pdf]

Data Sharing Statement

Chapel. Long-Term Health Improvements and Economic Performance Among Individuals With Diabetes. *JAMA Health Forum*. Published May 16, 2025.
doi:10.1001/jamahealthforum.2025.0756

Data
Data available: No

Additional Information
Explanation for why data not available: All data are publicly available from https://nhis.ipums.org/nhis/ and https://www.cdc.gov/brfss/annual_data/annual_data.htm; analytic code is available from the authors upon request.
